# Transcription factor GTF2I regulates osteoclast differentiation through mediating miR‐134‐5p and MAT2A expressions

**DOI:** 10.1002/ccs3.70010

**Published:** 2025-04-03

**Authors:** Lian Tang, Yanshi Liu, Jiyuan Yan, Lin Yuan, Zhaojun Wang, Zhong Li

**Affiliations:** ^1^ Department of Orthopedics Affiliated Hospital of Southwest Medical University Luzhou Sichuan China; ^2^ Department of Clinical Skills Center Affiliated Hospital of Southwest Medical University Luzhou Sichuan China; ^3^ Southwest Medical University Luzhou Sichuan China; ^4^ Stem Cell Immunity and Regeneration Key Laboratory of Luzhou Affiliated Hospital of Southwest Medical University Luzhou Sichuan China

**Keywords:** GTF2I, MAT2A, miR‐134‐5p, osteoclast differentiation, OVX

## Abstract

This study explored the possible effect of transcription factor GTF2I on the differentiation of osteoclasts and its regulation on the miR‐134‐5p/MAT2A axis. RANKL‐induced osteoclasts were measured for expressions of GTF2I, miR‐134‐5p, and MAT2A. The number and size of osteoclasts were assessed after TRAP staining. The expressions of osteoclast differentiation biomarkers, NFATC1, TRAP, and CTSK, were detected as well. The relationships of the GTF2I/miR‐134‐5p/MAT2A axis were verified by ChIP, dual luciferase, and RNA pull‐down assay. In vivo experiments were conducted on ovariectomized (OVX)‐treated mice to determine the effect of GTF2I overexpression on osteoclast differentiation and bone loss. RANKL‐induced osteoclasts had suppressed expressions of GTF2I and miR‐134‐5p and increased expression of MAT2A. GTF2I overexpression or miR‐134‐5p overexpression contributed to decreased cell number and size and suppressed cell differentiation, whereas such an effect can be abolished by overexpression of MAT2A. GTF2I can bind the miR‐134‐5p promoter to regulate its expression, whereas miR‐134‐5p can negatively regulate MAT2A expression. The protective effect of GTF2I overexpression against bone loss and cell differentiation was verified by in vivo experiments. Collectively, these results indicate that GTF2I can mediate miR‐134‐5p expression to increase MAT2A expression, contributing to the suppression of osteoclast differentiation.

## Graphical abstract

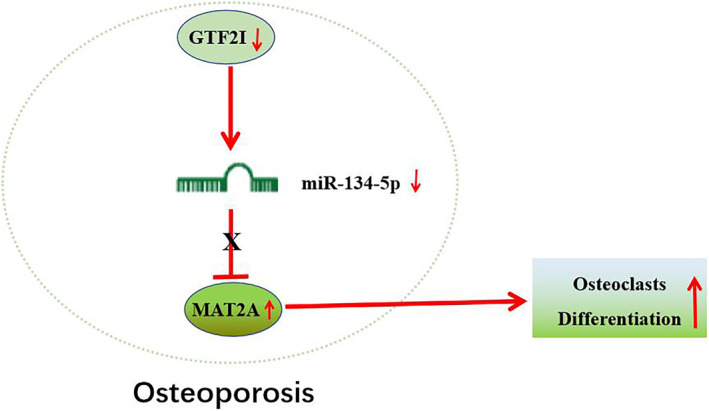

## 1 INTRODUCTION

Osteoclasts, originating from the monocytic lineage, play pivotal roles in bone shaping, calcium regulation, and hematopoiesis. They also contribute to osteoimmunity and the bone healing process under normal physiological circumstances.^1^, ^2^, ^3^ Maintaining a harmonious interplay between the bone resorption activities of osteoclasts and the bone formation processes of osteoblasts is essential for bone health.^4^, ^5^ An imbalance in this dynamic can trigger skeletal disorders that impact a vast demographic. When the coordination between osteoblast‐driven bone formation and osteoclast‐driven bone resorption is disrupted, it can lead to conditions such as postmenopausal osteoporosis and other secondary osteoporosis forms, including those linked with diabetes and glucocorticoid treatment.^6^, ^7^ Osteoporosis manifests as reduced bone mass and structural deterioration, which elevates the risk of fractures. Often dubbed a “silent disease,” osteoporosis typically does not present symptoms until a fracture occurs.^8^ Contemporary research is delving deeper into the molecular intricacies guiding osteoclast function in bone hemeostasis.^9^

First identified a quarter‐century ago, general transcription factor II‐I (GTF2I) emerged as a pivotal transcription factor with significant roles in diverse biological functions, notably in gene expression modulation.^10^ Current genome‐wide studies have linked GTF2I to at least three autoimmune disorders: primary Sjögren syndrome, systemic lupus erythematosus, and rheumatoid arthritis.^11^, ^12^ *GTF2I* gene is situated on chromosome 7, in a segment commonly deleted in Williams–Beuren syndrome. This connection suggests that GTF2I might be a potential gene of interest for WBS, a condition marked by developmental anomalies, including craniofacial structures and stature, hinting at disrupted bone development.^13^ Interestingly, GTF2I suppression was observed to enhance *ALPL* and *BGLAP* expression during the initial phases of osteoblast differentiation in C2C12 mouse myoblasts.^14^ Previous evidence also demonstrated GTF2I as a potential novel regulator in osteogenesis.^15^ However, GTF2I's precise involvement in osteoclast differentiation remains to be elucidated.

Research consistently indicates that miRNAs play a role in a range of biological processes, including osteoclastogenesis.^16^ By targeting essential molecules integral to osteoclast development, miRNAs can regulate bone resorption. Specifically, miR‐134‐5p is not only recognized for inhibiting tumor invasion but is also associated with neurological disorders, such as depression and Alzheimer's disease. Recent findings highlighted miR‐134‐5p′s role in promoting calcium accumulation in vascular smooth muscle cells of rats, suggesting its potential involvement in matrix deposition, a process crucial for bone development.^17^, ^18^ Notably, when miR‐134‐5p is suppressed, there is a marked increase in osteoclast formation and cell proliferation, coupled with reduced apoptosis.^19^

Our recent research identified the target relationship between miR‐134‐5p and MAT2A. Methionine adenosyltransferase 2A (MAT2A), a pivotal enzyme in the methionine cycle, predominantly facilitates the conversion of methionine and ATP into S‐adenosylmethionine (SAM). MAT2A's potential as a therapeutic target in cancer treatments has gained attention.^20^ Additionally, its essential role in osteoclastogenesis suggests that MAT2A may offer a promising therapeutic target for osteoporosis related to osteoclast dysfunction.

Despite much progress that has been made in the study of osteoclast differentiation in recent years, the exact relationship between GTF2I, miR‐134‐5p, and MAT2A remains unclear. In this study, we gained insight into the effect of GTF2I on osteoclast differentiation via the miR‐134‐5p/MAT2A signaling axis.

## 2 MATERIALS AND METHODS

### 2.1 Mice

Specific pathogen‐free (SPF) female C57BL/6 mice (*n* = 40, 6–8 weeks) were purchased from Hunan Silaike Jingda Laboratory Animal Co. Ltd. (Changsha, China) and housed in cages with a temperature of 21–25°C and humidity of 50%–65%. The light and dark cycle was set at 12 h/12 h. All mice had access to pellets and water. The use of animals was approved by the animal ethics committee of Southwest Medical University (Approval No. 20221028‐017). All animal experiments were carried out in a manner consistent with the *Regulations on the Management of Laboratory Animals*.

### 2.2 Extraction and culture of mouse bone marrow macrophages (BMMs)

Mice were anesthetized and euthanatized by cervical dislocation, and their bilateral ankles, knees, and hip joints of the lower limbs were collected. The tibias were obtained and placed in a phosphate‐buffered saline (PBS)‐containing petri dish to remove residues from the surface. The distal and proximal parts of the tibias were removed to expose the marrow cavity, which was repeatedly washed with PBS using a 1‐mL syringe. The collected marrow was resuspended in α‐MEM (SH30265.01, Hyclone, USA) at 1500 r/min for 4 min with the supernatant abandoned. The cells were then resuspended in a complete culture medium that contains 30 ng/mL macrophage colony‐stimulating factor (M‐CSF) and then cultured in a 10‐cm petri dish overnight. The culture medium was removed the next day, and cells were washed with sterile PBS twice and cultured in a complete culture medium containing 30 ng/mL M‐CSF for 2 days. Once cells were presented in a short shuttle shape with a concentration of 80%–90%, the culture medium was removed. The cells were washed with PBS 3 times and digested with trypsin before cells being counted and used as BMMs.

### 2.3 Immunofluorescence (IF)

The digested cells were counted and placed on a plate for cell culture with 2 × 10^5^ cells per well. Cells with a concentration of 60%–80% were washed with PBS for 3 × 5 min before fixation using 4% paraformaldehyde for 15 min and treated with PBS‐diluted permeabilization solution with 1% Triton X‐100 on ice for 2 min. After that, the cells were washed again and reacted with 5% serum for blocking for 1h, followed by another round of PBS washing. The PBS‐prepared primary antibody of F4/80 (ab6640, 1:200, Abcam) was added for cell incubation and green fluorescence‐labeled FITC‐goat anti‐rabbit primary antibody (ab150077, 1:500) for incubation at room temperature in the dark for 1 h. DAPI was used for nuclear staining for 15 min, and images were captured under a fluorescence microscope (Olympus IX51, Tokyo, Japan) under the same exposure condition.

### 2.4 BMMs induced for osteoclast differentiation

BMMs were placed in a 24‐well plate at a concentration of 1 × 10^5^ cells per well and cultured overnight before being induced for osteoclast differentiation using α‐MEM complete culture medium + 50 ng/mL receptor activator of nuclear factor κB ligand (RANKL) + 30 ng/mL M‐CSF. The culture medium was replaced every 2 days. On the fourth day of cell culture, BMMs were converged and cells with more than 3 nuclei were observed. Mature osteoclasts in a pancake shape with clear boundaries were observed under the microscope on the sixth day of cell culture.

### 2.5 Cell transfection

GTF2I overexpression vector (oe‐GTF2I) and its negative control (oe‐NC), miR‐134‐5p inhibition vector (in‐miR‐134‐5p) and inhibitor NC (in‐NC), miR‐134‐5p overexpression vector (miR‐134‐5p mimic) and mimic NC, and MAT2A overexpression vector (oe‐MAT2A) and its negative control (oe‐NC) were purchased from HanBio Technology (Shanghai, China). The viral titer was measured using a p24 ELISA kit (Cell Biolabs Inc., San Diego, USA) after the vectors were transfected into 293T cells for 48 h. The vectors were then transfected into BMMs for 24 h, and the cells were further cultured for 48 h to screen the stably transfected cell lines using puromycin (P8230, Beijing Solarbio Science and Technology Co. Ltd.). The transfection efficiency was tested before the following experiments were conducted.

### 2.6 Reverse transcription‐quantitative polymerase chain reaction (RTq‐PCR)

TRIzol (Invitrogen, Carlsbad, CA, USA) was used to extract total RNA from tissues or cells. The cDNA was obtained using a reverse transcription kit (TaKaRa, Tokyo, Japan) for mRNA or the miRNA First Strand cDNA Synthesis (Tailing Reaction) kit (B532451‐0020, Sangon, Shanghai, China) for miRNA. All operations were conducted based on instructions. SYBR Green Mix (Takara) was applied in the Applied Biosystems 7300 Real‐Time PCR System (ABI, Foster City, CA, USA). The reaction condition includes predenature at 95ºC for 5 min, denature at 95ºC for 10 s, annealing at 56ºC for 10 s, and extension at 72ºC for 20 s, 40 cycles in total. The expression of target genes was related to that of GAPDH or U6 using the 2^−ΔΔCt^ method .^21^ The primer sequences are listed in Table 1.

**TABLE 1:** Primer sequences for reverse transcription‐quantitative polymerase chain reaction.

Name of primer	Sequences (5′‐3′)
GTF2I‐F	GTTATCCCTGGGCTTCGTCC
GTF2I‐R	ACTCTTCATCTTCGGCAGGC
miR‐134‐5p‐F	TATGTGACTGGTTGACCAGAGGGG
miR‐134‐5p‐R	GTGCAGGGTCCGAGGT
MAT2A‐F	GTCTGTGCAGGAGGGTTCTT
MAT2A‐R	GTTTCCCCTCACGAGAAGTC
NFATc1‐F	GGTGCCTTTTGCGAGCAGTATC
NFATc1‐R	CGTATGGACCAGAATGTGACGG
TRAP‐F	CCGTGTTCCTACCCCCAATG
TRAP‐R	GGTCTCCTGGAACCTCTTGT
CTSK‐F	CTTCCAATACGTGCAGCAGA
CTSK‐R	TTGCATCGATGGACACAGAG
U6‐F	CTCGCTTCGGCAGCACA
U6‐R	AACGCTTCACGAATTTGCGT
GAPDH‐F	AGCCCAAGATGCCCTTCAGT
GAPDH‐R	CCGTGTTCCTACCCCCAATG

*Note*: F, forward; R, reverse.

### 2.7 Western blot

Cells or tissues were lysed on ice using RIPA lysis buffer (Beyotime, Shanghai, China), followed by centrifugation to collect the lysate and obtain protein samples. The concentration of proteins was measured using the BCA Kit (Beyotime). a certain volume of protein was mixed with loading buffer (Beyotime) for heating in a boiling water bath for 3 min to achieve protein denaturation. After that, the proteins were subjected to electrophoresis at 80 V for 30 min and 120 V for 90 min before transferring to membranes for 100 min with the current at 250 mA. The membranes were washed for 1–2 min before blocking them at 4°C overnight. On the next day, the primary antibodies of GTF2I (PA5‐17642, 1:1000, Thermo Fisher), MAT2A (ab177484, 1:1000, Abcam), CTSK (ab187647, 1:1000, Abcam), TRAP (ab52750, 1:1000, Abcam), NFATC1 (#4389, 1:1000, Cell Signaling Technology), and GAPDH (ab8226, 1:5000, Abcam) were added for incubation in a shaking table for 1 h. The membranes were washed for 3 × 10 min and further incubated with secondary goat anti‐rabbit IgG (ab6702, 1:5000, Abcam) or goat anti‐mouse IgG (ab6708, 1:5000, Abcam) for 1 h, followed by washing for 3 × 10 min. The membranes were reacted with a color‐developing solution and analyzed using a chemiluminescence imaging system (Bio‐Rad, Hercules, CA, USA).

### 2.8 Detection of differentiation ability of osteoclasts

The viability and bone resorption of osteoclasts were detected using the TRAP staining kit (Sigma‐Aldrich, St. Louis, MO, USA). Once mature osteoclasts in pancake shape with clear boundaries were observed on the sixth day of differentiation, the culture mediums of the 24‐well plates were removed and cells were fixed with 4% paraformaldehyde for 10 min after PBS washing. Cells were then washed in PBS twice and reacted with TRAP staining based on instructions in the kit. Cells were photographed and observed under a microscope (Olympus). TRAP positive cells with nuclei ≥3 were considered osteoclasts. ImageJ was used for quantitative analysis.

### 2.9 Dual‐luciferase reporter gene assay

TransmiR (http://www.cuilab.cn/transmir) predicted the binding sites of miR‐134‐5p with GTF2I, and starBase (https://masysu.com/) predicted the binding sites of miR‐134‐5p with MAT2A. According to the predictions, the mutant and wild‐type sequences of the binding sites were designed and inserted into the pGL3‐promoter vector for co‐transfection with oe‐GTF2I, miR‐134a‐5p mimic (30 nM), or a negative control into BMMs. About 48 h after cell transfection, the Dual‐Luciferase Reporter Assay System (Promega, Madison, WI, USA) and a spectrophotometer (Turner BioSystems, USA) were used to detect the luciferase activity.

### 2.10 Chromatin immunoprecipitation (ChIP) assay

The binding of GTF2I with miR‐134‐5p was verified using the EZ‐Magna ChIP TMA Kit (17‐10086, Millipore, USA). BMMs at the logarithmic growth period were crosslinked with 1% formaldehyde for cell culture for 10 min, and this process was terminated using 125 mM glycine for 5 min at room temperature. Cells were washed with precooled PBS twice and centrifuged at 2000 g for 5 min. The collected cells were suspended in cell lysis to make cell concentration 2 × 10^6^ cells per 200 μL and then mixed with a protease inhibitor mixture for centrifugation at 5000 g for 5 min. Cells were resuspended in a nucleus isolation buffer and lysed in an ice water bath for 10 min. Cells were sonicated into chromatin fragments of 200–1000 bp and centrifuged at 14,000 g at 4°C for 10 min to collect the supernatant. The supernatant (100 μL, DNA fragment) in each group was mixed with 900 μL ChIP dilution buffer, 20 μL 50 × PIC and 60 μL protein A agarose/salmon sperm DNA for 1 h at 4°C. The mixture was maintained at 4°C for 10 min before centrifugation at 700 g for 1 min. The supernatant was collected with 20 μL supernatant used as input. The supernatant in the experimental group was reacted with 1 μL GTF2I antibody (PA5‐17642, 1:100, Thermo Fisher) whereas the supernatant in the negative control group was reacted with 1 μL anti‐rabbit IgG (ab172730, 1:100, Abcam). 60 μL protein A agarose/salmon sperm DNA were added to both groups for reaction at 4°C for 2 h, followed by maintaining for 10 min and centrifugation at 700 g for 1 min. The supernatant was removed, and the sediments were washed with 1 mL low‐salt buffer, high‐salt buffer, LiCl solution, and TE solution (twice) before the sediments were eluted with 250 μL ChIP wash buffer per tube. Then, 20 μL of 5M NaCl was used for de‐cross‐linking and the DNA was obtained for PCR detection.

## 3 RNA PULL‐DOWN

Cell lysates isolated from BMMs, which were mixed with 100 ng of a biotin‐labeled NC probe or miR‐134‐5p probe, were incubated with the cell lysate at 25°C for 2 h. The complex was captured using streptavidin‐labeled magnetic beads at 25°C for 1 h and cultured in buffer containing protease K for 1 h at 25°C before being eluted and further detected using qRT‐PCR.

### 3.1 Ovariectomized (OVX) mouse models

Forty C57BL/6 female mice were equally grouped into OVX, sham, OVX + soe‐GTF2I, and OVX + oe‐NC groups. The OVX models were established based on methods described in a previous study.^22^ Pentasorbital sodium (0.3%, 0.1 mL/10 g) was used as an anesthetic for mice via intraperitoneal (ip) injection. The mice were shaved and disinfected before a 1.5‐cm incision was made in the middle line of the back. The bilateral ovaries and fallopian tubes were exposed through the abdominal cavity after the muscles of the ribcage and lumbar vertebrae were separated. Mice in the OVX group had their ovaries and fallopian tubes removed, whereas mice in the sham group were removed with a certain amount of adipose tissues around the bilateral ovaries. Mice were checked for active bleeding, and the incision was closed. Penicillin was given to mice for 3 days postoperatively to prevent infection. Mice in the OVX + oe‐NC group or OVX + oe‐GTF2I group were injected with oe‐NC or oe‐GTF2I lentiviruses into the tail vein. The concentration of the lentiviruses was 1 × 10^9^ TU/mL. About 4 weeks after the operation, femoral tissues were collected for further detection .^22^, ^23^

### 3.2 Hematoxylin and eosin (H&E) staining

Tissues were fixed in 4% paraformaldehyde for 72 h before embedding and slicing. The slices were then dehydrated in gradient alcohol and permeabilized with xylene before washing in deionized water, staining with hematoxylin for 3–5 min, washing in deionized water, and differentiation with hydrochloric acid alcohol for 20 s. After that, slices were treated with 1% ammonia for 30 s and restained with 1% eosin solution for 5 min. Slices were washed after each process using either running water or deionized water. The prepared slices were routinely hydrated, permeabilized, dried, and sealed before observation under a microscope.

### 3.3 Micro‐CT analysis

The left tibia of the mouse was collected with soft tissues removed for scanning under the micro‐CT system (PerkinElmer, Japan) at 90 kV and 88 μA. The parameters were set as follows: a resolution of 4000 × 2672, an aluminum sheet of 0.2 μm, and a thickness of 10 μm. The cancellous bone of the proximal tibia was analyzed, and the following indexes were detected: bone volume/tissue volume (BV/TV), bone mass density (BMD), trabecular thickness (Tb.Th), trabecular number (Tb.N), and trabecular segregation (Tb.Sp).

### 3.4 Statistical analysis

GraphPad Prism 8.0 was used for data analysis with all data expressed as mean ± standard deviation. A *t*‐test was used for comparison between two groups, whereas one‐way analysis of variance and Tukey's multiple comparisons test were used for comparison among multiple groups. The statistical significance was set at a *p* value less than 0.05.

## 4 RESULTS

### 4.1 GTF2I overexpression suppresses osteoclastogenesis

**FIGURE 1:**
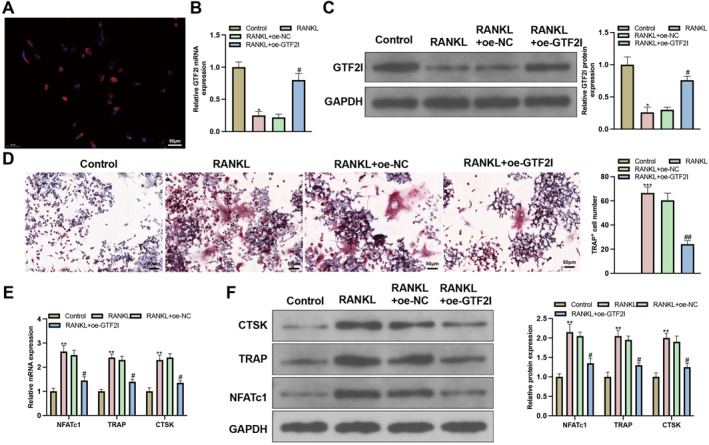
GTF2I overexpression restricts osteoclastogenesis. (A) BMMs biomarker F4/80 was detected by immunofluorescence (*N* = 3); (B‐C) RT‐qPCR and western blot detected the mRNA and protein expressions of GTF2I (*N* = 3); (D) osteoclastogenesis was observed using TRAP staining (*N* = 3); (E‐F) RT‐qPCR and western blot detected the mRNA and protein expressions of NFATC1, TRAP, and CTSK (*N* = 3). Data were expressed as mean ± standard deviation. Comparisons among multiple groups were conducted using one‐way ANOVA followed by Tukey's multiple comparisons test for post‐hoc analysis. Cellular experiments were repeated 3 times. *p < 0.05, **p < 0.01, ***p < 0.001 when compared with the control group; ^#^ *p* < 0.05, ^##^ *p* < 0.01 when compared with the RANKL + oe‐NC group. BMMs, bone marrow macrophages.

BMMs from mice were extracted and identified with immunofluorescence for positive expression of F4/80 (Figure 1A). Then, the BMMs were induced by M‐CSF and RANKL before the expression of GTF2I was detected. The results showed inhibited GTF2I expression in the M‐CSF + RANKL group in contrast to that in the control group (Figure 1B,C). The oe‐GTF2I vector was transfected into cells in the M‐CSF + RANKL group, and the detection showed that the oe‐GTF2I vector could significantly increase GTF2I expression (Figure 1B,C). The cell morphology of BMMs before and after transfection was observed and compared. TRAP staining demonstrated that the RANKL group had more cell number and size compared with the control group, but such an observation was reversed in cells after oe‐GTF2I transfection when compared with cells transfected with oe‐NC (Figure 1D). The detection of osteoclast biomarkers NFATC1, TRAP, and CTSK by qRT‐PCR and western blot showed that the expression of these biomarkers was increased in response to RANKL treatment but further decreased in response to oe‐GTF2I transfection (Figure 1E,F). These results showed that GTF2I was decreasingly expressed in osteoclasts, whose overexpression restricts osteoclastogenesis.

### 4.2 Transcription factor GTF2I promotes GTF2I expression to mediate osteoclastogenesis

**FIGURE 2:**
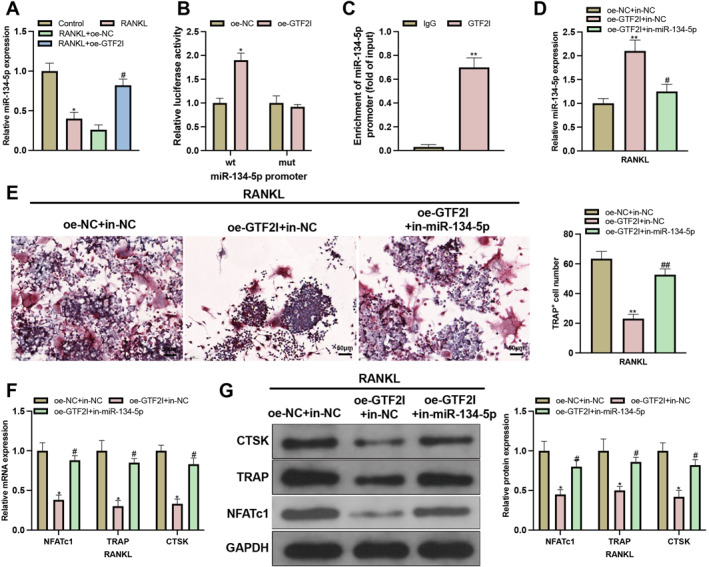
GTF2I promotes GTF2I expression by binding its promoter to mediate osteoclastogenesis. (A) RT‐qPCR detected the expression of miR‐134‐5p (*N* = 3); (B‐C) ChIP and dual‐luciferase reporter gene assay confirmed the binding of GTF2I with miR‐134‐5p (*N* = 3); (D) after cell transfection, RT‐qPCR detected the expression of miR‐134‐5p (*N* = 3); (E) osteoclastogenesis was assessed using TRAP staining (*N* = 3); (F‐G) after cell transfection, the expressions of NFATC1, TRAP, and CTSK were detected by RT‐qPCR and western blot (*N* = 3). Data were expressed as mean ± standard deviation. Comparisons between two groups were conducted using the *t‐*test, whereas comparisons among multiple groups were conducted using one‐way ANOVA followed by Tukey's multiple comparisons test for post‐hoc analysis. Cellular experiments were repeated 3 times. *p < 0.05, **p < 0.01 when compared with the control, oe‐NC, IgG, or oe‐NC + in‐NC group; ^#^ *p* < 0.05, ^##^ *p* < 0.01 when compared with the oe‐GTF2I + in‐NC group.

Although decreased GTF2I expression was found in osteoclasts, how GTF2I regulates osteoclastogenesis remains to be determined. TransmiR predicted the binding sites of GTF2I with the miR‐134‐5p promoter. Considering the function of miR‐134‐5p in osteoclasts, we speculated that GTF2I might bind the miR‐134‐5p promoter to mediate its miRNA expression. To verify this hypothesis, we detected miR‐134‐5p expression, and the results showed that miR‐134‐5p expression was decreased in the RANKL group, whereas oe‐GTF2I transfection could increase miR‐134‐5p expression (Figure 2A). The binding of GTF2I with miR‐134‐5p was verified by a ChIP and dual‐luciferase reporter gene assay, which demonstrated that oe‐GTF2I can significantly increase the luciferase activity of the wild‐type sequence, instead of the mutant type (Figure 2B,C). These results confirmed the binding and regulation of GTF2I on miR‐134‐5p.

oe‐GTF2I and in‐miR‐134‐5p were co‐transfected into BMMs (Supplementary Figure 1A). The detection of miR‐134‐5p expression showed that cells transfected with oe‐GTF2I had increased miR‐134‐5p expression compared with the oe‐NC+in‐NC group, whereas cells co‐transfected with oe‐GTF2I and in‐miR‐134‐5p had suppressed miR‐134‐5p expression in contrast to cells co‐transfected with oe‐GTF2I and in‐NC (Figure 2D). The results of osteoclastogenesis indicated that compared to the oe‐GTF2I + in‐NC group, the oe‐GTF2I + in‐miR‐134‐5p group had increased number and size of osteoclasts, as well as elevated expression levels of the osteoclast marker genes NFATc1, ACP5, and CTSK (Figure 2E–G).

### 4.3 miR‐134‐5p negatively mediates MAT2A expression

**FIGURE 3:**
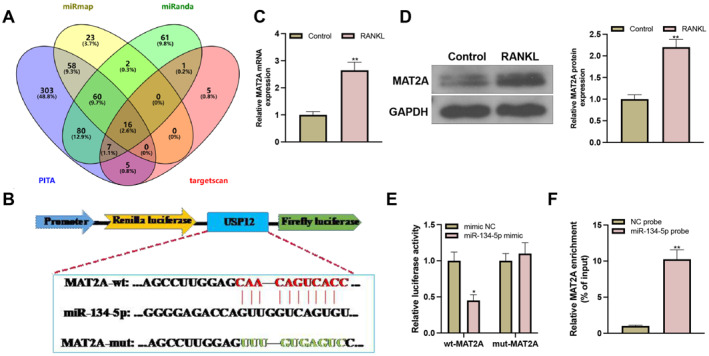
miR‐134‐5p negatively regulates MAT2A expression. (A) the mRNAs that can bind miR‐134‐5p were predicted; (B) binding sites of miR‐134‐5p with MAT2A; (C‐D) MAT2A mRNA and protein expressions were detected by RT‐qPCR or western blot (*N* = 3); (E‐F) RNA pull‐down and dual‐luciferase reporter gene assay confirmed the binding of miR‐134‐5p with MAT2A (*N* = 3). Data were expressed as mean ± standard deviation. Comparisons between two groups were conducted using the *t‐*test. Cellular experiments were repeated 3 times. *p < 0.05, **p < 0.01 when compared with the control, mimic NC, or NC probe group.

PITA, miRmap, miRanda, and TargetScan predict the mRNAs that can bind miR‐134‐5p. The overlaps of the mRNAs are shown in Figure 3A, among which MAT2A was predicted to bind miR‐134‐5p (Figure 3B). The suppression of MAT2A expression can inhibit osteoclastogenesis. Therefore, we speculated that MAT2A may work as a downstream target of miR‐134‐5p in osteoclastogenesis. To confirm this hypothesis, RT‐qPCR or western blot was applied to detect the expression of MAT2A. The measurement showed that MAT2A was increasingly expressed in the RANKL group compared with the control group (Figure 3C,D). A dual‐luciferase reporter gene assay showed that miR‐134‐5p mimic could significantly suppress the luciferase activity of wt‐MAT2A but not mut‐MAT2A (Figure 3E), which was consistent with the results of the RNA pull‐down assay (Figure 3F). Those results supported the negative regulation of miR‐134‐5p on MAT2A expression.

### 4.4 miR‐134‐5p suppresses MAT2A expression to inhibit osteoclastogenesis

**FIGURE 4:**
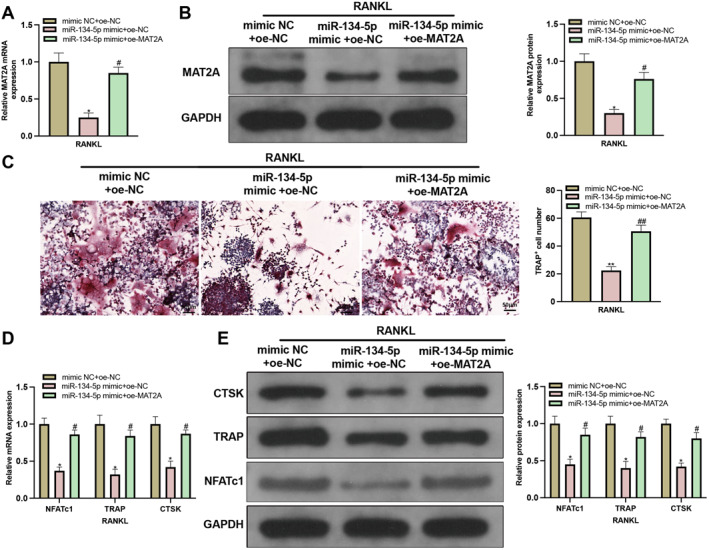
miR‐134‐5p inhibits osteoclastogenesis through mediating MAT2A expression. (A‐B) RT‐qPCR and western blot detected MAT2A expression (*N* = 3); (C) osteoclastogenesis was assessed using TRAP staining (*N* = 3); (D‐E) NFATC1, TRAP, and CTSK expressions were detected using RT‐qPCR and western blot (*N* = 3). Data were expressed as mean ± standard deviation. Comparisons among multiple groups were conducted using one‐way ANOVA followed by Tukey's multiple comparisons test for post‐hoc analysis. Cellular experiments were repeated 3 times. *p < 0.05, **p < 0.01 when compared with the mimic NC + oe‐NC group. ^#^ *p* < 0.05, ^##^ *p* < 0.01 when compared with the miR‐134‐5p mimic + oe‐NC group.

miR‐134‐5p mimic or/and oe‐MAT2A were transfected into M‐CSF + RANKL‐treated BMMs, in which the expression of MAT2A was detected (Supplementary Figure 1B‐C). The results showed that MAT2A expression was suppressed in response to miR‐134‐5p mimic transfection but rebounded after further transfection with oe‐MAT2A (Figure 4A,B). The detection of MAT2A expression in cells transfected with in‐miR‐134‐5p showed elevated MAT2A expression in the in‐miR‐134‐5p group compared to the in‐NC group (Supplementary Figure 2A‐B). The assessment on osteoclastogenesis showed that miR‐134‐5p mimic could suppress osteoclastogenesis but such suppression could be abolished by co‐transfection with oe‐MAT2A and miR‐134‐5p mimic (Figure 4C–E).

### 4.5 The effect of GTF2I overexpression on osteoclast differentiation can be reversed by MAT2A overexpression

**FIGURE 5:**
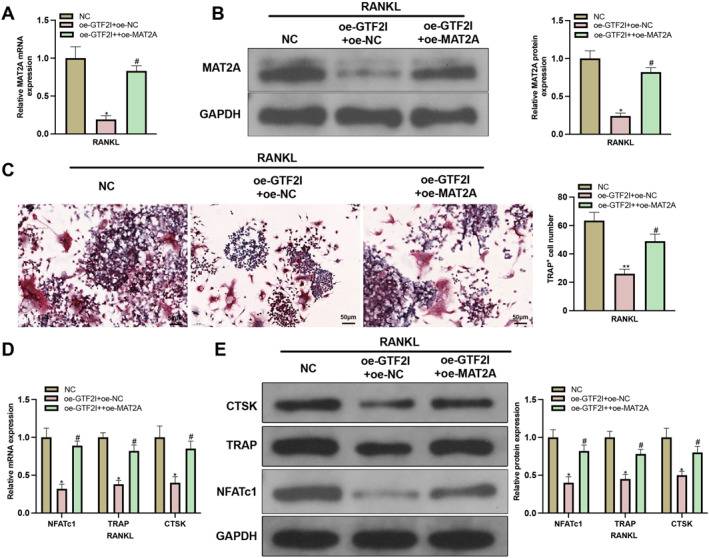
MAT2A can abolish the suppressive effect of GTF2I overexpression on osteoclast differentiation. (A‐B) RT‐qPCR and western blot detected MAT2A expression (*N* = 3); (C) TRAP staining observed osteoclastogenesis (*N* = 3); (D‐E) RT‐qPCR and western blot detected the expressions of NFATC1, TRAP, and CTSK (*N* = 3); data were expressed as mean ± standard deviation. Comparisons among multiple groups were conducted using one‐way ANOVA followed by Tukey's multiple comparisons test for post‐hoc analysis. Cellular experiments were repeated 3 times. *p < 0.05, **p < 0.01 when compared with the NC group. ^#^ *p* < 0.05, ^##^ *p* < 0.01 when compared with the oe‐GTF2I + oe‐NC group. The NC group was set for both the oe‐GTF2I + oe‐NC group and oe‐GTF2I + oe‐MAT2A group.

miR‐134‐5p mimic or/and oe‐MAT2A were transfected into M‐CSF + RANKL‐treated BMMs. The changes in MAT2A expression were assessed, and the results showed that oe‐GTF2I alone could significantly inhibit MAT2A expression, which could be reversed by oe‐MAT2A (Figure 5A–B). The measurement on miR‐134‐5p expression revealed that oe‐GTF2I can promote miR‐134‐5p expression, but further transfection with oe‐MAT2A showed no significant change in miR‐134‐5p expression (Supplementary Figure 2C). TRAP staining presented that oe‐MAT2A could reverse the suppressive effect of oe‐GTF2I on osteoclast differentiation to further promote osteoclastogenesis (Figure 5C). A similar expression pattern was found in the detection of osteoclast biomarkers (Figure 5D,E). GTF2I mediates osteoclast differentiation of BMMs via the miR‐134‐5p/MAT2A axis in vitro.

### 4.6 GTF2I mediates osteoporosis via the miR‐134‐5p/MAT2A axis in vivo

OVX mouse models were established in which oe‐GTF2I was injected. RTq‐PCR and western blot demonstrated that compared with the ham group, the OVX group had decreased miR‐134‐5p expression and increased MAT2A expression in the femoral tissues, but elevated expressions of miR‐134‐5p and GTF2I, as well as decreased MAT2A expression, were found in the femoral tissues of OVX mice with oe‐GTF2I treatment (Figure 6A,B). H&E staining showed that femur tissues and trabecular bone were arranged orderly with less trabecular breakage and a normal‐sized bone cavity, whereas the condition in the OVX group was much worse with more bone and tissue separation, trabecular breakage, and an increased bone cavity, which could be attenuated by oe‐GTF2I (Figure 6C). The micro‐CT scan showed that intact trabecular bone in the sham group, but the Tb.N and bone loss increased significantly, and significant changes in bone surface area and volume were found in the OVX group (Figure 6D). The OVX group also had decreased BV/TV, BMD, Tb.Th, Tb.N, cortical area (Ct.Ar), and cortical thickness (Ct.Th) and increased Tb.Sp when compared with the sham group, and such a condition could be improved after oe‐GTF2I treatment (Figure 6E–K). The detection of NFATC1, TRAP, and CTSK showed that TRAP, CTSK, and NFATC1 mRNA and protein expressions were increased in the OVX group in contrast to the sham group but suppressed in response to oe‐GTF2I treatment (Figure 6L,M). These results showed that GTF2I mediates osteoporosis in mice through regulating the miR‐134‐5p/MAT2A axis.

FIGURE 6GTF2I mediates osteoporosis in mice through regulating the miR‐134‐5p/MAT2A axis. (A‐B) RTq‐PCR and western blot detected GTF2I, miR‐134‐5p, and MAT2A expressions (*N* = 10); (C) H&E staining for morphological observation (*N* = 10); (D‐K) micro‐CT analyze the bone microenvironment, BV/TV, BMD, Tb.Th, Tb.N, Tb.Sp, Ct.Ar, and Ct.Th (*N* = 10); (L‐M) RT‐qPCR and western blot detected expressions of NFATC1, TRAP, and CTSK (*N* = 10); data were expressed as mean ± standard deviation. Comparisons among multiple groups were conducted using one‐way ANOVA followed by Tukey's multiple comparisons test for post‐hoc analysis. *p < 0.05, **p < 0.01, when compared with the sham group. ^#^ *p* < 0.05 when compared with the OVX + oe‐NC group. Tb.Th, trabecular thickness; BMD, bone mass density; BV/TV, bone volume/tissue volume; Tb.N, trabecular number; Tb.Sp, trabecular segregation; Ct.Ar, cortical area; Ct.Th, cortical thickness. Abbreviation: BMM, bone marrow macrophages; RTq‐PCR, reverse transcription‐quantitative polymerase chain reaction.

**Figure:**
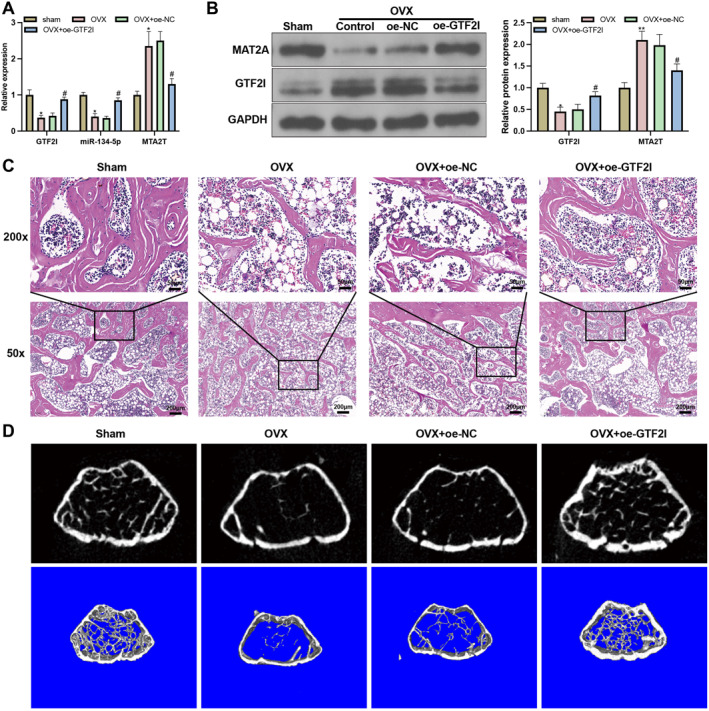

**Figure:**
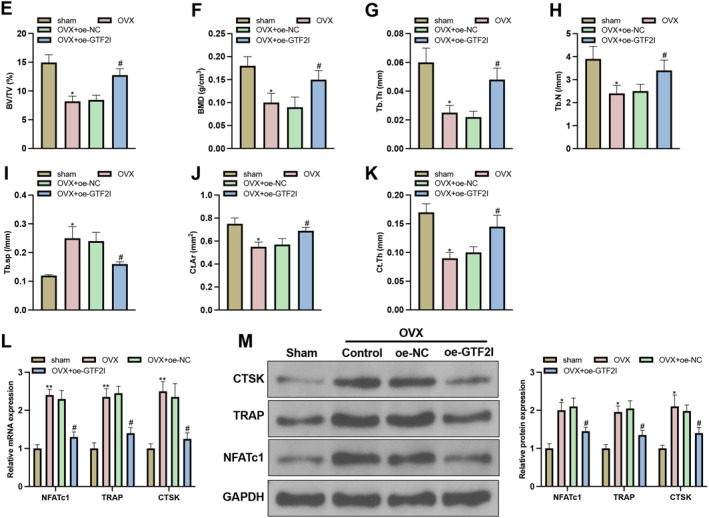

## 5 DISCUSSION

Osteoclast differentiation is a central process in bone health and disease, and its imbalance can lead to a variety of bone diseases, such as osteoporosis .^24^ In recent years, with the development of molecular biology techniques, more and more studies have begun to explore the molecular mechanisms affecting osteoclast differentiation .^25^ Among these mechanisms, transcription factors often play a key role in influencing cell function by regulating the expression of specific genes. GTF2I is a known transcription factor, but its role in osteoclast differentiation has not been fully studied. In this study, we demonstrated that the transcription factor GTF2I promoted MAT2A expression by regulating the levels of miR‐134‐5p, affecting osteoclast differentiation.

Osteoporosis, an important topic in the study of bone health, is caused by an imbalance in the activity of osteoclasts and bone‐forming cells .^26^ Osteoblast differentiation from mesenchymal stem cells stands as a cornerstone in bone development and maintenance. This intricate process, when derailed, can predispose individuals to debilitating conditions, notably bone cancer or osteoporosis .^27^ In our comprehensive exploration, we discerned a marked diminution in the expression of the transcription factor GTF2I, particularly in OVX mice and osteoclasts. This observation accentuated the findings of a prior investigation that elucidated the indispensable role of GTF2I in the orchestration of osteogenesis .^15^ Although the multifaceted functionalities of GTF2I span across diverse biological realms, its concrete implication in preserving bone health remains an enigmatic domain, warranting further probing.

In the process of an in‐depth exploration of how GTF2I affects osteoclast differentiation, we found a complex regulatory relationship between GTF2I and miR‐134‐5p. Specifically, as a transcription factor, GTF2I promoted the expression of miR‐134‐5p to suppress osteoclastogenesis. miRNAs serve as intricate modulators, steering the course of chondrogenic and osteogenic differentiation. Their influence is exerted by selectively targeting genes or transcription factors that either promote or inhibit these processes .^28^ Among these, miR‐134‐5p has emerged as a significant player, based on a growing body of evidence. It has been implicated in a spectrum of cellular biological activities and metabolic pathways, notably influencing cellular invasion capabilities and metastatic propensities .^29^, ^30^, ^31^ Moreover, this miRNA affects the dynamics and progression of various cancers, including breast, colorectal, and ovarian .^32^, ^33^, ^34^ An intriguing observation from an earlier study spotlighted the negative association of miR‐134 with osteosarcoma (OS)'s aggressive tendencies, correlating with heightened metastatic inclinations and a hastened mortality rate .^35^ Consistently, suppressing miR‐134‐5p promoted osteoclast development, whereas its augmentation hindered it .^19^

miRNAs play a pivotal role in orchestrating osteoclast differentiation by exerting inhibitory control over specific downstream genes .^36^ Within the scope of our research, we employed the dual‐luciferase reporter assay to ascertain the potential target genes influenced by miR‐134‐5p. Both mRNA and protein levels of MAT2A confirmed its direct regulation by miR‐134‐5p. To decipher MAT2A's role in osteoclastogenesis, we exposed M‐CSF and RANKL‐treated BMMs to miR‐134‐5p mimic, either alone or combined with oe‐MAT2A. Our findings demonstrated that the miR‐134‐5p mimic independently hindered osteoclastogenesis, but its combination with oe‐MAT2A counteracted this effect, promoting BMM osteoclast formation. Beyond our study, MAT2A has garnered attention as a promising target in cancer therapeutics .^20^ A concurrent study showcased that the targeted pharmacological blockade of MAT2A using AG‐270, or its genetic inhibition via MAT2A‐shRNA, led to a pronounced reduction in osteoclast formation and its associated functions in vitro .^37^ Corroborating our findings, Xia et al. highlighted an appreciable surge in MAT2A levels in OS samples. They further posited that miR‐26b‐5p exerted its anticancer prowess by acting upstream of MAT2A, subsequently diminishing its expression within OS tumor entities .^38^ Our study also verified that GTF2I affected osteoporosis in mice through the miR‐134‐5p/MAT2A axis.

In summary, we identified a new signaling axis: GTF2I/miR‐134‐5p/MAT2A, which plays a vital role in osteoclast differentiation. Specifically, GTF2I promotes the expression of miR‐134‐5p to reduce MAT2A expression, thus suppressing osteoclastogenesis. Our study provides a unique perspective to understand the regulatory mechanisms of bone health. Although we have verified the interactions between these molecules through a variety of experimental methods, further research is needed to confirm the biological significance of these results.

## AUTHOR CONTRIBUTIONS

LZ and TL conceived the ideas; designed the experiments. TL; LYS; YYJ; YL and WZJ performed the experiments. LYS; YYJ; YL and WZJ analyzed the data. TL and LYS provided critical materials. YL and WZJ wrote the manuscript. LZ supervised the study. All the authors have read and approved the final version for publication.

## CONSENT FOR PUBLICATION

Not applicable.

## CONFLICT OF INTEREST STATEMENT

The authors declare there is no conflicts of interest.

## ETHICS STATEMENT

The use of animals was approved by the animal ethics committee of Southwest Medical University (Approval No. 20221028‐017). All animal experiments were carried out in a manner consistent with the “Regulations on the Management of Laboratory Animals”.

## Supporting information

Supplementary Information S1

Figure S1

Figure S2

## Data Availability

The datasets used or analyzed during this study are available from the corresponding author upon reasonable request.
