# Supplementary material for: Transcription factor GTF2I regulates osteoclast differentiation through mediating miR‐134‐5p and MAT2A expressions
Source: J Cell Commun Signal. 2025 Apr 3;19(2):e70010. doi: 10.1002/ccs3.70010 (PMC11968177; doi:10.1002/ccs3.70010)
Supplement: Supplementary file 1 — Supplementary Information S1 [file CCS3-19-e70010-s001.docx]

**Supplementary Figure 1. Validation of transfection efficiency**

**A-C, RTq-PCR detected miR-134-5p and MAT2A expressions. Data were expressed as mean ± standard deviation. Comparisons between two groups were conducted using the T-test. Cellular experiments were repeated 3 times. ***P < 0.001, when compared with in-NC, mimic NC or oe-NC groups.**

Supplementary Figure 2. Validation of transfection efficiency

A-B, RTq-PCR and western blot detected MAT2A expressions; C, RTq-PCR detected miR-134-5p expression. Data were expressed as mean ± standard deviation. Comparisons among multiple groups were conducted using one-way ANOVA, followed by Tukey’s multiple comparisons test for post hoc analysis. Cellular experiments were repeated 3 times. ***P < 0.001, when compared with in-NC, oe-GTF2I+oe-NC groups.
