# Supplementary figures and images for: Transcription factor GTF2I regulates osteoclast differentiation through mediating miR‐134‐5p and MAT2A expressions
Source: J Cell Commun Signal. 2025 Apr 3;19(2):e70010. doi: 10.1002/ccs3.70010 (PMC11968177; doi:10.1002/ccs3.70010)

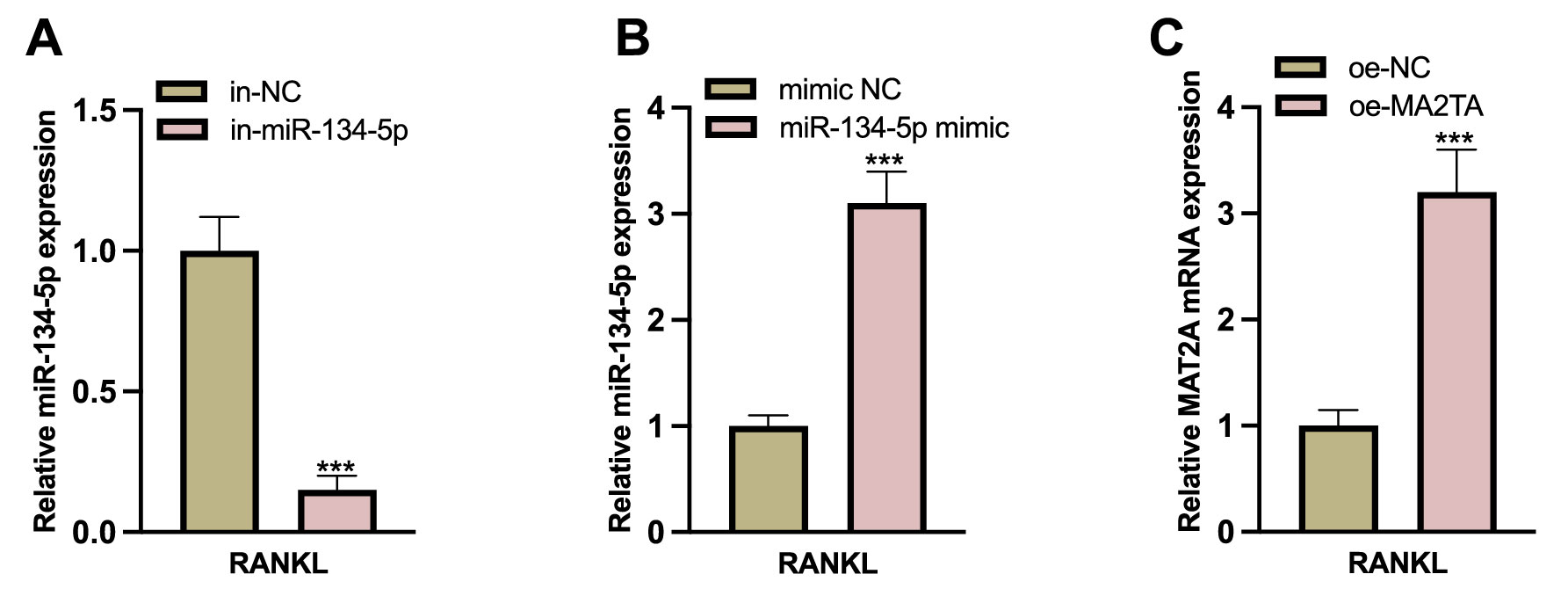

Supplement: Supplementary file 2 — Figure S1 [file CCS3-19-e70010-s002.jpg]

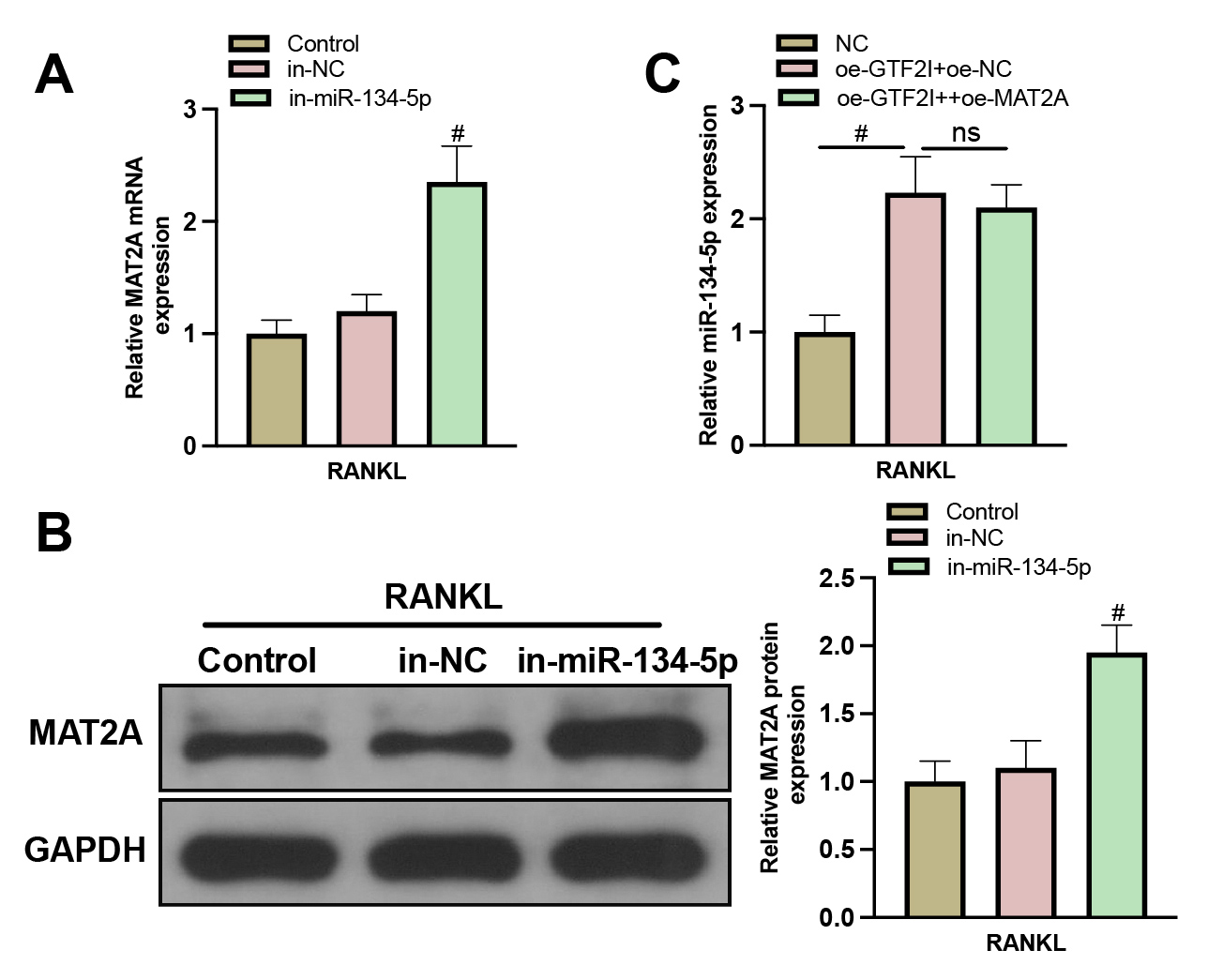

Supplement: Supplementary file 3 — Figure S2 [file CCS3-19-e70010-s003.jpg]
